# False Positive for Malaria Rapid Test in a Patient With Cytomegalovirus Infection

**DOI:** 10.7759/cureus.63896

**Published:** 2024-07-05

**Authors:** Andrés E Prieto-Torres, Abraham Katime Zuñiga, Bertha Lacouture Ortiz, Álvaro A Faccini-Martínez

**Affiliations:** 1 Internal Medicine, Hospital Militar Central, Bogotá, COL; 2 Internal Medicine, Clinica El Prado, Santa Marta, COL; 3 Bacteriology, Universidad del Magdalena, Santa Marta, COL; 4 Infectious Disease Service, Hospital Militar Central, Bogotá, COL; 5 Faculty of Medicine, Universidad Militar Nueva Granada, Bogotá, COL; 6 Services and Consulting in Infectology, Infectious Disease Service, Bogotá, COL

**Keywords:** cytomegalovirus, false positive, rapid immunochromatographic tests, colombia, malaria infection

## Abstract

There is a growing development of immunochromatographic tests for the detection of specific *Plasmodium* spp. antigens. These tests rely on capturing antigens from peripheral blood using monoclonal or polyclonal antibodies against specific targets. We present the case of a 28-year-old male patient with a history of two previous episodes of *Plasmodium falciparum *malaria, treated appropriately seven months and three years ago. He was referred to our institution with a six-day history of fever, epigastric pain, hematuria, and vomiting. Serial thick and thin blood smears were negative for hemoparasites, but a Bioline™ Malaria Ag P.f/Pan rapid test was positive for the Pan (pLDH) band. Given the clinical context and inability to visualize *Plasmodium* in blood smears, the positive pLDH band on the rapid malaria test was considered a possible false positive. Subsequent tests concluded that the patient was experiencing a cytomegalovirus (CMV) infection, which improved with supportive management, and he was discharged symptom-free. Malaria remains a major public health issue in tropical and subtropical regions. While rapid diagnostic tests are crucial for timely diagnosis, false positives due to cross-reactivity with other infections and conditions are reported. Our case highlights the potential for cross-reactivity with CMV infections, although direct evidence of active viral replication was not obtained. This phenomenon can lead to the overestimation of malaria cases and inappropriate treatment, underscoring the need for careful interpretation of rapid test results.

## Introduction

People living in tropical regions of the world are significantly exposed to a variety of infectious and non-infectious diseases with similar clinical presentations. Some infectious diseases have various diagnostic methods available, while others are diagnosed based on clinical symptoms through the exclusion of other more probable causes. Therefore, the available diagnostic tests must guarantee adequate sensitivity and specificity to ensure that patients are being treated for the true cause of their illness [1]. This is particularly important in a tropical country like Colombia, where tropical infections such as malaria constitute a current public health issue [2]. The low specificity of diagnostic tests can lead to an overestimation of malaria cases and the overuse of antimalarial drugs.

Although direct examination of peripheral blood through blood smears and thick smears has long been considered the gold standard for diagnosing malaria infections, in many places, there is no availability of trained personnel to perform these types of tests [3]. There is an increasing development of immunochromatographic tests for the detection of specific parasite antigens.

The principle of these tests is based on the capture of antigens from peripheral blood using monoclonal or polyclonal antibodies against specific targets. Currently, these tests can detect *Plasmodium falciparum* histidine-rich protein 2 (a soluble protein produced by the parasite that remains expressed on the surface of erythrocytes for up to 28 days after the initiation of antimalarial therapy), *Plasmodium* aldolase (an enzyme of the glycolytic pathway expressed in the blood stages of all parasitic species), and parasitic lactate dehydrogenase (a glycolytic enzyme produced by both sexual and asexual stages and released from parasitized erythrocytes, common to all species) [4].

Although there is increasing evidence in the literature of false positives in rapid diagnostic tests, mostly in patients with positive rheumatoid factor and diseases such as Chagas disease, American trypanosomiasis, and salmonella [1], to date, we have not found case reports related to false positives with cytomegalovirus (CMV) infections in immunocompetent patients.

We report the case of a young patient diagnosed with CMV infection, whose rapid test results came back positive with the Bioline™ Malaria Ag P.f/Pan test. This test has demonstrated a sensitivity and specificity of 99.5% and 99.7%, respectively, for the HRP2 antigen and 95.5% and 99.5%, respectively, for the pLDH antigen [5].

## Case presentation

The patient is a 28-year-old male, currently an active-duty military member, originally from Plato, Magdalena, with a recent travel history to Tumaco, Nariño (within the last 30 days prior to admission). Relevant medical history includes previous episodes of *Plasmodium falciparum* malaria infections treated appropriately, seven months and three years ago. He was referred from a primary care facility to our institution in Barranquilla, Colombia, due to a six-day history of fever, reaching temperatures as high as 40°C, along with epigastric pain, hematuria, vomiting, and melenas. Initial tests from the referring facility showed severe thrombocytopenia. The patient was admitted with a suspected diagnosis of dengue virus infection (as per the written report from the referring facility, indicating a positive IgM test). Subconjunctival hemorrhage was noted on the physical examination at admission. Given the severity of thrombocytopenia and hemorrhagic symptoms, the patient was transferred to the intensive care unit. Institutional tests revealed a negative rapid dengue test, a hemogram showing a tendency towards neutropenia with relative lymphocytosis, without anemia but severe thrombocytopenia, hyperbilirubinemia due to indirect bilirubin elevation, elevated lactate dehydrogenase, normal liver transaminases, and normal coagulation times. Tests for rheumatoid factor and hepatitis C and B virus infections were all negative, CMV antibody tests were positive, and a thick smear for malaria was negative (Table 1). The patient's condition improved significantly after four days of monitoring and supportive care, intravenous isotonic fluid therapy, and antipyretics with acetaminophen. He was subsequently transferred to the general internal medicine ward and showed complete improvement with the resolution of bleeding and normalization of platelet count and was discharged on the seventh day.

**Table 1:** Laboratory reports

Parameter	Results	Control (two weeks later)	Reference values
P24 antigen HIV	Negative	-	-
Leukocytes	4.7	2.65	4-10 10^9^/L
Neutrophils	1.79	0.79	2-7 10^9^/L
Lymphocytes	2.59	1.59	0.8-4 10^9^/L
Hemoglobin	12.1	8.8	12-16 gr/dL
Hematocrit	33	25	40-54%
Platelets	48	162	150-450 10^9^/L
Total bilirubin	3.7	1.3	0.3-1.2 mg/dL
Indirect bilirubin	2.9	0.9	0.2-0.8 mg/dL
Lactate dehydrogenase	727	420	125-220 U/L
Cytomegalovirus IgG	94	122	Positive: >14 U/mL. Negative: <12 U/mL
Cytomegalovirus IgM	37	34	Positive: >22 U/mL. Negative: <18 U/mL
Dengue IgM-IgG-NS1	Negative	-	-
Toxoplasma IgG	64	-	Positive: >10 UA/mL. Negative: <10 UA/mL
Toxoplasma IgM	6.9	-	Positive: >8 UA/mL. Negative: <6 UA/mL

However, the patient was readmitted to the institution seven days later, reporting persistent fevers since discharge, mainly occurring in the evening, ranging between 38 and 39°C, without any other associated symptoms. He had sought medical care at a dispensary 72 hours ago, where they reported a positive rapid malaria test. Laboratory results this time showed marked bicytopenia, primarily affecting the white blood cell line, and anemia. Bilirubin levels, transaminases, and renal function were within normal ranges, and lactate dehydrogenase levels were decreasing. Given the patient's clinical context and the inability to visualize the parasite in direct examinations, the positive pLDH malaria test was considered a possible false positive. Serial thick smears were negative, and a follow-up CMV antibody test showed increasing IgG levels and decreasing IgM levels (Table 1). It was concluded that the patient was experiencing a CMV infection, which improved with supportive management, and he was finally discharged home without symptoms.

## Discussion

CMV infections are frequently asymptomatic or subclinical. There are three types of infection depending on the immune status of the individual. Individuals without prior exposure to the virus develop a primary infection upon initial contact. Reactivation of the virus after a period of latency, during which it remains in mononuclear cells without manifesting as a clinical disease, constitutes the second form of infection. The third category is reinfection, which occurs in previously infected individuals who develop the infection upon new contact with the virus, despite having natural immunity [6].

The clinical manifestations of CMV infection include myalgias, fever, malaise, headache, and fatigue, along with lymphocytosis and abnormal liver function tests. Less common manifestations include pharyngitis, lymphadenopathy, rash, splenomegaly, and hepatomegaly. Approximately 90% of primary infections in immunocompetent individuals are asymptomatic, occasionally resulting in a self-limited mononucleosis-like illness lasting 3-4 weeks. There are isolated reports of severe primary infections with multi-organ involvement in these patients. Immunocompromised individuals may develop complications such as hemolytic anemia, enteritis, transverse myelitis, colitis, thrombocytopenia, and encephalitis. Reactivations typically occur in immunosuppressed patients, especially those with HIV infection with low CD4 counts and patients undergoing solid organ and hematopoietic stem cell transplants [7].

Diagnosing CMV infection is complex. For systemic infections, polymerase chain reaction (PCR) is the gold standard for detecting the virus in blood. Serology is not specific enough to classify an infection as acute or chronic; it merely suggests prior exposure and should not be used as a diagnostic method. Therefore, evidence from cultures or immunohistochemistry showing typical cytoplasmic inclusions constitutes definitive proof of the disease [8].

In our patient, while we lack isolation of the virus or evidence of organ-specific infection through immunohistochemistry, the clinical presentation is highly suggestive. This includes initial gastrointestinal involvement and cytopenias that improved with supportive care alone. Serological tests in this case are not diagnostic but indicate a reactivation or reinfection of CMV, suggesting a probable CMV infection in the absence of more definitive tests. The key point of this report is that after a thorough examination, we found no cause clearly associated with the false-positive result of the rapid malaria diagnostic test. The interval between previous infections and the current episode also does not explain the result, making it plausible to associate the false-positive result with a probable CMV infection.

Malaria remains a major public health problem in tropical and subtropical regions. The development of rapid diagnostic tests represents a significant advancement in the timely diagnosis of febrile patients. However, there is a growing body of literature reporting false positives in patients with positive rheumatoid factor, antinuclear antibodies, and rapid plasma reagin tests [9], as well as infections such as hepatitis C, toxoplasmosis, trypanosomiasis, dengue, leishmaniasis, Chagas disease, and schistosomiasis [10,11]. These infections can produce soluble proteins that cross-react with the antibodies used in the tests. Additionally, errors in sample processing, exposure to extreme temperatures, and humidity can alter results [12].

Cross-reactivity phenomena can lead to an overestimation of malaria cases and inappropriate use of antimalarials, resulting in failure to timely diagnose and treat the true underlying conditions. To date, there have been no reported cases of cross-reactivity of these rapid tests with CMV infections. Although we lack evidence of active viral replication by molecular tests or histological confirmation of tissue involvement in our patient, the clinical presentation is highly suggestive of CMV infection.

## Conclusions

This case report underscores the complexity and diagnostic challenges of infectious diseases in regions where malaria is endemic. It is crucial to recognize the potential for cross-reactivity in rapid diagnostic tests, as misdiagnosis can lead to inappropriate treatment and a failure to address the true underlying conditions.

This publication is significant as it draws attention to an unreported interaction between CMV infections and malaria rapid diagnostic tests, contributing valuable insights to the existing body of medical literature. It underscores the importance of comprehensive diagnostic approaches and the need for healthcare professionals to consider alternative diagnoses in patients presenting with febrile illnesses, particularly in tropical and subtropical regions.

By enhancing understanding of the limitations and cross-reactivity of current diagnostic tools, this report advocates for the development of more specific and sensitive diagnostic methods. Ultimately, this will improve patient outcomes, prevent unnecessary treatments, and promote better management of infectious diseases in endemic areas.
